# A prospective study of oral 5-aminolevulinic acid to prevent adverse events in patients with localized prostate cancer undergoing low-dose-rate brachytherapy: Protocol of the AMBER study

**DOI:** 10.1016/j.conctc.2020.100593

**Published:** 2020-06-17

**Authors:** Makito Miyake, Nobumichi Tanaka, Isao Asakawa, Kaori Yamaki, Takashi Inoue, Shota Suzuki, Shunta Hori, Yasushi Nakai, Satoshi Anai, Kazumasa Torimoto, Michihiro Toritsuka, Hitoshi Nakagawa, Shinji Tsukamoto, Tomomi Fujii, Chiho Ohbayashi, Masatoshi Hasegawa, Masato Kasahara, Kiyohide Fujimoto

**Affiliations:** a Department of Urology, Nara Medical University, 840 Shijo-cho, Kashihara, Nara, 634-8522, Japan; b Departmen of Prostate Brachytherapy, Nara Medical University, 840 Shijo-cho, Kashihara, Nara, 634-8522, Japan; c Department of Radiation Oncology, Nara Medical University, 840 Shijo-cho, Kashihara, Nara, 634-8522, Japan; d Institute for Clinical and Translational Science, Nara Medical University, 840 Shijo-cho, Kashihara, Nara, 634-8522, Japan; e Department of Psychiatry, Nara Medical University, 840 Shijo-cho, Kashihara, Nara, 634-8522, Japan; f Department of Cardiovascular Medicine, Nara Medical University, 840 Shijo-cho, Kashihara, Nara, 634-8522, Japan; g Department of Orthopedic Surgery, Nara Medical University, 840 Shijo-cho, Kashihara, Nara, 634-8522, Japan; h Department of Diagnostic Pathology, Nara Medical University, 840 Shijo-cho, Kashihara, Nara, 634-8522, Japan

**Keywords:** Prostate cancer, Urinary frequency, Low-dose-rate brachytherapy, Radiotherapy, Radioprotection, 5-Aminolevulinic acid, Adverse event, ALA, 5-aminolevulinic acid, CTCAE, Common Toxicity Criteria for Adverse Events, EBRT, extra-beam radiotherapy, EPIC, Expanded Prostate Cancer Index Composite, GI, gastrointestinal, GU, genitourinary, IPSS, International Prostate Symptom Score, I-125, iodine-125, J-POPS, Japanese nationwide prospective cohort study, LDR-BT, low-dose-rate brachytherapy, OABSS, overactive bladder symptom score, PCa, prostate cancer, PRO, patient reported outcome, PSA, prostate-specific antigen, QOL, quality of life, SFC, sodium ferrous citrate, SHIM, Sexual Health Inventory for Men

## Abstract

**Background:**

Radiotherapy is one of the most frequently selected treatment options for patients with prostate cancer. However, adverse effects related to the irradiated surrounding normal organs are significant clinical concerns. Specifically, genitourinary and gastrointestinal toxicities can lead to a dramatically reduced quality of life. The aim of this clinical trial is to determine the efficacy of oral 5-aminolevulinic acid (ALA) phosphate with sodium ferrous citrate (SFC) in patients treated with low-dose-rate brachytherapy (LDR-BT) using an iodine-125 seed source.

**Methods:**

The AMBER study is a prospective, single-center trial in patients with localized prostate cancer undergoing LDR-BT. Patients who undergo supplementary extra-beam radiotherapy are excluded, whereas those who undergo pre-implantation short-term (4–6 months) androgen deprivation therapy to decrease the prostate volume and/or improve oncological outcomes are included. After the screening and registration, the patients will be instructed to take capsules of ALA-SFC twice a day (200 mg and 229.42 mg per day) for 6 months from the day of seed implantation (prescribed radiation dose of 160 Gy). Patient data will be collected before the implantation; during oral ALA-SFC treatment; and 1, 3, 6, 9, and 12 month(s) after seed implantation. The primary endpoint of this trial is the urinary frequency 3 months after seed implantation. At each visit, the 24-h urinary frequency, total voided volume, and mean voided volume on a frequency volume chart and other patient-reported outcomes are recorded. The data of the trial cases will be compared with those of historical controls, who are consecutive patients undergoing LDR-BT without supplementary extra-beam radiotherapy between January 2016 and January 2019. The number of subjects has been set to be 50 for trial cases and 150 for the historical control cases. Pre- and post-treatment clinicopathologic factors are compared between two groups.

**Discussion:**

The goal of this trial is to determine the potential benefit of ALA-SFC in patients who undergo LDR-BT. To the best of our knowledge, this is the first study investigating the potential clinical benefit of oral ALA-SFC after radiotherapy. More evidence from a further randomized controlled trial is needed to change the standard of care and lead to better post-radiotherapy management.

**Trial registration:**

This clinical trial was prospectively registered with the Japan Registry of Clinical Trials on 5 December 2019. The reference number is jRCTs051190077, nara0013 (Certified Review Board of Nara Medical University).

## 1 Introduction

Radiotherapy is one of the most frequently selected treatment options for patients with prostate cancer (PCa). According to a Japanese nationwide prospective cohort study (J-POPS), thousands of patients undergo low-dose-rate brachytherapy (LDR-BT) using an iodine-125 (I-125) seed source per year [1]. Currently, brachytherapy is a well-established treatment modality for localized PCa in terms of organ preservation and preferable long-term oncological outcomes [2]. However, adverse effects related to the irradiated surrounding organs are significant clinical concerns in the management after implantation. Specifically, the gastrointestinal (GI) and genitourinary (GU) complications can lead to a dramatically reduced quality of life (QOL) [[3], [4], [5]].

Studies over the past several decades have investigated the pathophysiological and molecular mechanisms underlying radiotherapy-induced effects on both tumor tissue and normal tissue [6,7]. Although several pharmacological interventions that potentially prevent radiation-induced damage in normal tissues have been explored in preclinical studies, only a couple of them have progressed to clinical use [6]. Our previous study was the first to demonstrate the dual benefit of supplementary oral 5-aminolevulinic acid (ALA) and pelvic radiotherapy in a syngenic PCa model, i.e., MyC-CaP cells in FVB mice [7]. The “dual benefit” includes radiosensitization of PCa tumor tissues and radioprotection of normal pelvic organs from radiotherapy. ALA is distributed ubiquitously in mammalian cells and is a precursor of porphyrins and heme protein, which play essential roles in aerobic energy metabolism and the electron transport system [8]. Preclinical studies have demonstrated that ALA can confer a broad range of cytoprotective effects against cisplatin-induced nephrotoxicity, rhabdomyolysis-induced acute kidney injury, hypoxia-induced cardiomyocyte injury, and neurotoxicity in preclinical studies [[9], [10], [11], [12]]. Based on the results, an interventional single-arm clinical trial is ongoing to evaluate whether oral ALA phosphate with sodium ferrous citrate (ALA-SFC) prevents cisplatin-induced renal injury in patients with unresectable gastric cancer (trial ID: UMIN000024642). Moreover, Japanese double-blinded, randomized, placebo-controlled trials are ongoing to investigate the clinical benefit of oral ALA-SFC for patients with mitochondrial disease (JMA-IIA00358), Alzheimer disease (jRCTs041180135), and autism spectrum disorder (jRCTs051190017).

This prospective, open-label, single-center, single-arm pilot trial evaluates the short-term toxicity, such as urinary frequency, and other types of complications of LDR-BT. The intervention in this trial is oral administration of ALA-SFC, which is widely accepted as a health supplement and food with functional claims. Based on the results obtained in this trial, we will design a double-blinded, randomized, placebo-controlled trial to consolidate the clinical value of this intervention. To the best of our knowledge, this is the first study investigating the potential clinical benefit of oral ALA-SFC after radiotherapy and is expected to lead to better post-radiotherapy management.

## 2 Materials and methods

### 2.1 Inclusion criteria, patient recruitment, and study design

**Fig. 1:**
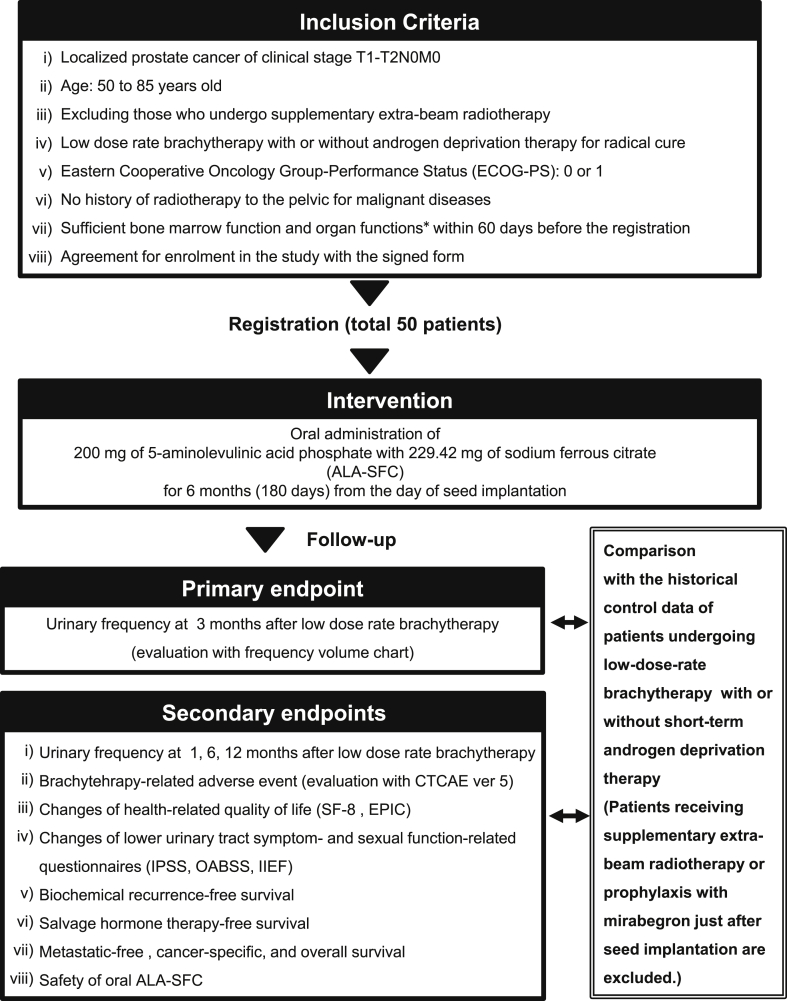
Design of the AMBER study: Inclusion criteria and endpoints. Patients should fulfill the indicated inclusion criteria to be eligible for this trial. * hemoglobin, ≥9.0 g/dL; white blood cell count, ≥12,000/mm^3^; absolute neutrophil count, ≥2000 cells/mm^3^; platelet count, ≥100,000 cells/mm^3^; normal kidney and liver functions as determined by creatinine, total bilirubin, aspartate transaminase (AST), and alanine transaminase (ALT) ≤2 × the upper limit of normal for the reference laboratory.

**Fig. 2:**
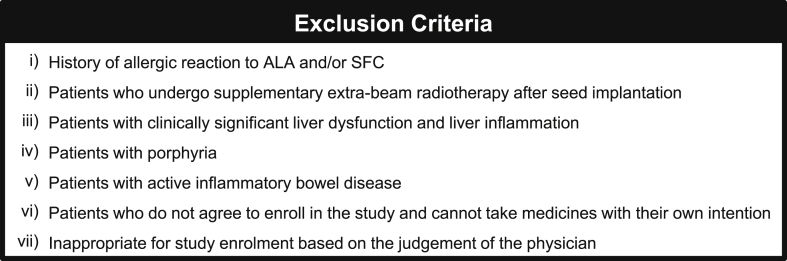
Exclusion criteria of the AMBER study. Abbreviations: ALA, aminolevulinic acid; SFC, sodium ferrous citrate.

This prospective, single-center trial in patients with localized PCa is ongoing at Nara Medical University (a Japanese academic hospital). The trial design and protocol adhere to the Recommendations for Interventional Trials (SPIRIT) criteria. The completed SPIRIT checklist can be found in Supplementary data. Investigators and patients will be aware of the intervention of ALA-SFC. The flowchart, inclusion criteria, primary endpoint, and secondary endpoints are depicted in Fig. 1. Patients who undergo supplementary extra-beam radiotherapy (EBRT) are excluded, whereas those undergoing short-term (4–6 months) neoadjuvant androgen deprivation therapy (ADT) to decrease the prostate volume and/or improve oncological outcomes are included. As a general rule of our hospital, the combination of luteinizing hormone-releasing hormone agonist/antagonist and oral bicalutamide (80 mg/day) are used for neoadjuvant ADT. The exclusion criteria are listed in Fig. 2. Informed consent and written consent forms of patients are mandatory before study participation. After the screening and registration, the patients will be enrolled to a single treatment group of a fixed dose of oral ALA-SFC (200 mg and 229.42 mg per day). Subjects will undergo the seed implantation under spinal anesthesia and be instructed to take capsules of ALA-SFC twice a day for 6 months from the day of seed implantation.

The use of α1-adrenoceptor antagonists to ameliorate post-seed urinary discomfort or prevent urinary retention is permitted. Prohibited medications and procedures are not set up in this trial. Medications before and after seed implantation should be recorded.

### 2.2 Procedure of LDR-BT

As of November 30, 2019, we have an LDR-BT treatment experiment of more than 1400 patients. The detailed procedure of the seed implantation has been previously described [13,14]. All the patients are hospitalized for 3 nights and 4 days. The prescribed radiation dose is 160 Gy for the monotherapy. For patients without any contraindications, SpaceOAR™ hydrogel is injected between the rectum and prostate to minimize high-dose radiation to the rectum and consequent bowel side effects. Experienced radiation oncologists (I. Asakawa and K. Yamaki) calculate the post-implant dosimetric parameters based on a pelvic computed tomography scan performed about 1 month after LDR-BT.

### 2.3 Follow-up, data collection, and data protection

**Fig. 3:**
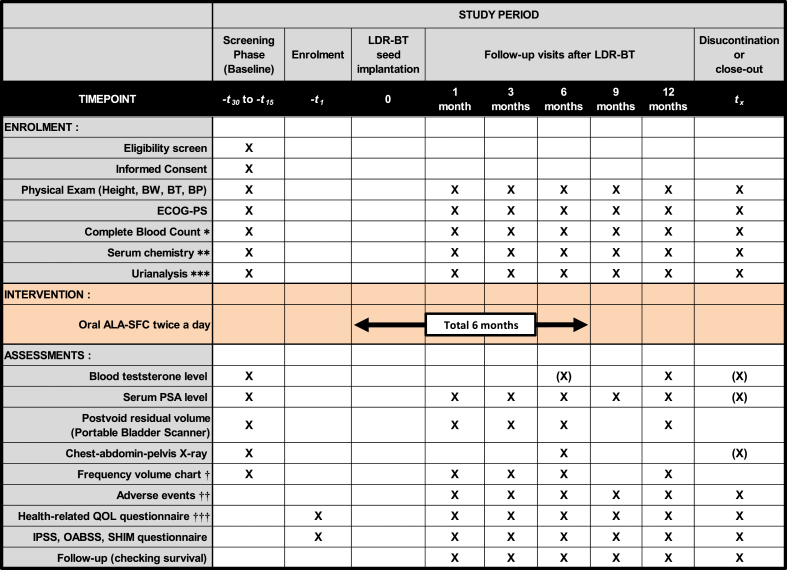
Intervention and assessment schedule of the AMBER study according to the Recommendations for Interventional Trials (SPIRIT). Full physical examinations are required at screening and every visit. Follow-up visits and data collection should occur approximately 1, 3, 6, 9, and 12 months from the seed implantation. Patients will complete a set of questionnaires at every visit, and follow-up information may be collected via medical charts. In case of information being unavailable in chart reviews, patients may be contacted via telephone or in person. Subject identification cards may be provided after the informed consent process and prior to seed implantation. The Case Report Form will include information regarding past history, concomitant medications, and any medications taken after the treatment. Chest–abdomen–pelvis X-ray will be performed to verify seed migration. X, mandatory; (X), optional. * Hemoglobin, hematocrit, white blood cell count and fractions, platelet count; ** aspartate transaminase (AST), alanine transaminase (ALT), g-glutamyl transpeptidase (g-GTP), total bilirubin, alkaline phosphatase (ALP), lactate dehydrogenase (LDH), total protein, albumin, serum creatinine, uric acid, total cholesterol, low-density lipoprotein (LDL)-cholesterol, high-density lipoprotein (HDL)-cholesterol, triglyceride, C-reactive protein (CRP), calcium, phosphorus, potassium, chloride; *** Urine dipstick test (specific gravity, pH, protein, glucose, bilirubin, urobilinogen, ketone body, occult blood) and urine sediment test; ^†^ 3 days and nights record. Patients pick days that will be convenient for them to measure and record everything; ^††^ According to the Common Toxicity Criteria for Adverse Events (CTCAE v 5.0) translated to Japanese; ^†††^ Questionnaires: SF-8™ and Expanded Prostate Cancer Index Composite (EPIC). Abbreviations: LDR-BT, low-dose-rate brachytherapy; BW, body weight; BT, body temperature; BP, blood pressure; ECOG-PS, Eastern Cooperative Oncology Group-performance status scale; PSA, prostate-specific antigen; QOL, quality of life; IPSS, International Prostate Symptom Score; OABSS, overactive bladder symptom score; SHIM, Sexual Health Inventory for Men.

**Fig. 4:**
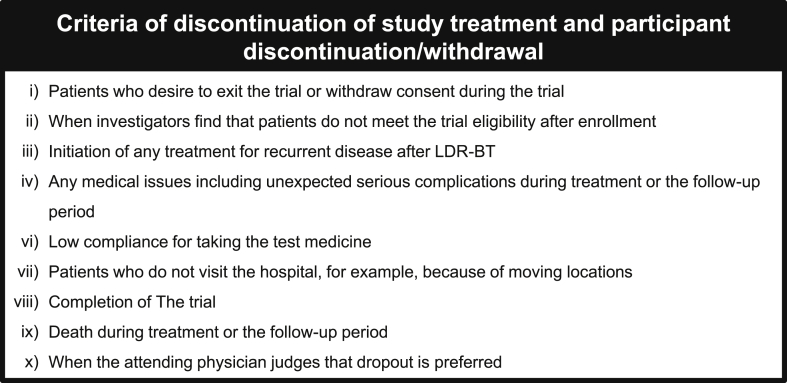
Discontinuation/dropout criteria of the AMBER study. Abbreviations: LDR-BT, low-dose-rate brachytherapy.

Patient data will be collected before the implantation; during oral ALA-SFC treatment; 1, 3, 6, 9, and 12 months after the implantation; and at the end of the trial (Fig. 3). After the initiation of this trial, we provide a specific diary booklet to the participants, by which they record taking the intervention drug. At each visit, the 24-h urinary frequency, total voided volume, and mean voided volume are recorded on a frequency volume chart (FVC) over 3 days [15]. The assessment includes patient reported outcomes (PROs) such as the SF-8™, Expanded Prostate Cancer Index Composite (EPIC), International Prostate Symptom Score (IPSS), overactive bladder symptom score (OABSS), and Sexual Health Inventory for Men (SHIM) questionnaire. The data will be documented in specific Case Report Forms regarding GU/GI, hematological, and other possible toxicities using the Common Toxicity Criteria for Adverse Events (CTCAE v 5.0), complete blood count, serum chemistry, and serum prostate-specific antigen (PSA) levels. The investigators will conduct assessments for potential new or worsening adverse events as indicated in the trial schedule and more frequently if clinically needed. Biochemical recurrence is defined as the Phoenix definition of a rise in PSA level by 2 ng/mL or more above the nadir PSA [16]. When clinical recurrence is suspected, imaging examinations, such as computed tomography, magnetic resonance imaging, and/or bone scans, are performed. After discontinuation or close-out of this trial, patients will be followed up every 6 months for 5 years and annually thereafter. Discontinuation/dropout criteria are listed in Fig. 4.

To protecting the patient data, unique identification codes will be provided to all patients. All the data will be protected in password-accessible files, and only investigators will be able to access the files. All data and documents will be deleted and discarded 5 years after the end of the trial unless the data are under secondary use for another study.

### 2.4 Determining the target sample size

To date, we have been working on several clinical studies regarding oncological outcomes, adverse events, complications, and changes in urinary frequency and PROs among patients undergoing LDR-BT [3,4,14,17,18]. In this study, the data of the trial cases will be compared with historical control data. Data of patients who underwent seed implantation monotherapy between February 2016 and February 2019 will be extracted for the historical control data. The primary endpoint of this trial is urinary frequency 3 months after the implantation. One of our previous studies showed that a significant increase in urinary frequency and significant decrease in mean voided volume persisted for 12 months after seed implantation [14]. The average times per day of urination increased from 9.2 at baseline to 12.2 at 3 months after seed implantation because of bladder irritability. This observation indicated that the increase in urinary frequency was 3 times per day at 3 months, which was the peak point. To determine the required sample size of this trial, we expect that oral ALA-SFC can suppress the increase in urinary frequency to only 1 time per day and estimate the standard deviation of urinary frequency to be 3.5 times both in trial cases and historical control cases. The ratio of case numbers is set to 1:3 for trial cases and historical control cases, respectively. Given the 10% proportion of ineligible patients, the required sample size was determined to be at least 37 for trial cases and 109 for historical control cases to provide 80% power (β = 0.20) and an α level of 0.05 (two-sided). Based on the estimation, a total of 50 trial cases and 150 historical control cases will be enrolled in this study. The historical data will be obtained from consecutive patients undergoing LDR-BT with or without short-term ADT between January 2016 and January 2019. Patients who received prophylaxis with 50 mg of mirabegron (a β3-adrenoceptor agonist) just after seed implantation for decreasing undesired urgency and urinary frequency will be excluded from the historical control cases. Patients who received supplementary extra-beam radiotherapy will be excluded from the historical control cases. Pre- and post-treatment clinicopathologic factors are compared between two groups: age, body mass index, comorbidities, pretreatment urinary frequency, pretreatment PSA level, pretreatment prostate volume, biopsy Gleason score, clinical tumor stage, D'Amico risk stratification, and the post-implant dosimetric parameters.

### 2.5 Interim analysis and monitoring

Because this is a short-term pilot study with a 12-month follow-up, we will not conduct interim analysis for the clinical efficacy of oral ALA-SFC. However, the safety of ALA-SFC will be independently evaluated by the Data and Safety Monitoring Committee at the time as follows:

i) Critical modification of the study protocol is required
ii) Any serious adverse event associated with this agent occurs
iii) A critical problem is observed while monitoring
iv) The principal investigator needs the judgement of this committee

Moreover, the monitoring committee independently evaluates whether the study is implemented in compliance with the study protocol, and the data are appropriately corrected according to a pre-arranged monitoring plan at the time when the first patients have been enrolled and once per year.

### 2.6 Statistical analysis

Descriptive statistics will be computed for all study variables. Comparison of the two groups, the trial cases and historical control cases, will be conducted using the Mann–Whitney *U* test or Wilcoxon signed rank test for continuous variables, and Fisher's exact test or Tarone test for categorical variables as appropriate. Survival analysis will be conducted using the Kaplan–Meier method and log-rank test. P < 0.05 will be considered statistically significant.

### 2.7 Trial registration

This clinical trial was prospectively registered with the Japan Registry of Clinical Trials on 5 December 2019. The reference number is jRCTs051190077, nara0013 (Certified Review Board of Nara Medical University). The URL of trial registry record is found in https://jrct.niph.go.jp/en-latest-detail/jRCTs051190077.

### 2.8 Dissemination

The results will be submitted for a peer-reviewed journals for publication and presented at local and international scientific conferences. Also, the results will be available to interested participants.

## 3 Discussion

It has been more than 15 years since LDR-BT was introduced in Japan [2]. A comprehensive review reported excellent oncological outcomes showing equivalent or superior efficacy to radical prostatectomy for low- or intermediate-risk PCa and superiority for high-risk PCa based on the experience of more than 52,000 patients undergoing LDR-BT with or without EBRT [2]. However, there are still significant concerns about side effects, complications, and consequently reduced QOL after radiotherapy. One of our long-term interests is worsening lower urinary tract symptoms. Most urinary adverse events are categorized into grade 1 to 2, while higher-severity adverse events are rare. More than 70% of patients experience increased urinary frequency (pollakiuria) during the first 6 months after seed implantation [19]. To overcome this clinical issue, our group and other groups have conducted randomized controlled trials to explore medications preventing post-treatment urinary adverse events. However, the true benefits of α1-adrenoceptor antagonists, anti-cholinergic drugs, β3-adrenoceptor agonists, phytotherapeutic drugs, and non-steroidal anti-inflammatory drugs have not been proven yet [14,[20], [21], [22]].

We plan to evaluate whether oral ALA-SFC prevents GU toxicities after seed implantation. ALA-SFC is widely accepted as a health supplement and food with functional claims in Japan. This product reportedly inhibits elevations in blood glucose level, improves sleep quality, and supports muscle strength and exercise efficiency [23,24]. Moreover, preclinical studies have demonstrated that ALA confers cytoprotective effects in the bladder, rectum, kidney, heart, and neural system from various types of stress [7,[9], [10], [11], [12]]. Another potential benefit of adding ALA is an increase in the radiosensitization of malignant cells. Previous research has shown that ALA supplementation sensitizes malignant cells including glioma, melanoma, and colon adenocarcinoma cells to radiotherapy via enhanced generation of protoporphyrin IX and reactive oxygen species [7]. Our previous preclinical study confirmed that adding *in vitro* single and *in vivo* repeated administration of ALA sensitized PCa cells to radiotherapy. Based on this evidence, this trial includes oncological outcomes such as biochemical recurrence-free survival as secondary endpoints.

Assessments based on the CTCAE are the gold standard for evaluating adverse events related to treatment. However, previous studies have demonstrated that agreement between the CTCAE assessment and PROs is poor to moderate [25]. Bennett et al. reported that the rate of patients reporting severe neuropathy is 30%, which is higher than that identified by the CTCAE assessment (10%) [26]. This suggests that toxicities are often undetected or underestimated by clinicians. This trial utilizes FVCs and PROs as well as the CTCAE for toxicity assessment. Our target primary endpoint in this trial is prevention of increasing urinary frequency after seed implantation. We suppose that the benefit of this intervention will be more precisely and objectively evaluated using FVCs.

The goal of this prospective, single-center trial is to determine the potential benefit of taking ALA-FSC in patients who undergo LDR-BT. We anticipate favorable control of GU toxicities and acceptable tolerance. The potential vast utility of ALA-SFC is further encouraging because this drug can sensitize cancer cells to radiotherapy. Ultimately, the promising results will provide patients with supplementary intervention and potentially changing the standard of care.

## Trial status

The study began in December 2019. Patient recruitment has not yet been completed and the intervention program is ongoing. A follow-up and data collection will be completed in March 2022. The final results are expected in September 2022.

## Ethics approval and consent to participate

This clinical trial complied with the Declaration of Helsinki regarding investigation in humans. Its ethical clearance, protocol (version 1.0 on November 11th, 2019), and associated documents were approved by the Certified Review Board of Nara Medical University (institution ID: CRB5180011). Informed consent and written consent forms of patients are mandatory before study participation.

## Consent for publication

Not required

## Availability of data and materials

The collected datasets used during this clinical trial are available from the corresponding author (N. Tanaka) on reasonable request.

## Authors' contributions

MM is the principal investigator and conceived the study. NT, MK, and KF are the advisors of the study and participated in the design and intervention of the study. IA, KY, SH, YN, SA, KT, and MH are major contributors of the data acquisition. TF and CO perform the histological examination of the prostate. SS and TI will statistically analyze and interpreted the patient data regarding the adverse events and other associated outcomes. MT, HN, and ST are major contributors of the monitoring and data protection. MM wrote the first draft of the manuscript and SS substantively revised it. All authors provided input into the study design, provided intellectual input to the manuscript and approved the final version of the manuscript.

## Funding

The AMBER study is funded by SBI Pharmaceuticals Co., Ltd. The role of the funding body is production and provision of the intervention drug (ALA-SFC).

## Declaration of competing interest

MM, NT, and KF have received a joint research fund from SBI Pharmaceuticals Co., Ltd, Tokyo, Japan, that produces ALA-SFC capsules and partially supported this clinical trial.

## References

[1] Saito S., Ito K., Yorozu A., Aoki M., Koga H., Satoh T., Ohashi T., Shigematsu N., Maruo S., Kikuchi T., Kojima S., Dokiya T., Fukushima M., Yamanaka H. (2015). Nationwide Japanese prostate cancer outcome study of permanent iodine-125 seed implantation (J-POPS). Int. J. Clin. Oncol..

[2] Tanaka N., Asakawa I., Hasegawa M., Fujimoto K. (2020). Low-dose-rate brachytherapy for prostate cancer: a 15-year experience in Japan. Int. J. Urol..

[3] Miyake M., Tanaka N., Asakawa I., Hori S., Morizawa Y., Tatsumi Y., Nakai Y., Inoue T., Anai S., Torimoto K., Aoki K., Hasegawa M., Fujii T., Konishi N., Fujimoto K. (2017). Assessment of lower urinary symptom flare with overactive bladder symptom score and International Prostate Symptom Score in patients treated with iodine-125 implant brachytherapy: long-term follow-up experience at a single institute. BMC Urol..

[4] Tanaka N., Asakawa I., Anai S., Hirayama A., Hasegawa M., Konishi N., Fujimoto K. (2013). Periodical assessment of genitourinary and gastrointestinal toxicity in patients who underwent prostate low-dose-rate brachytherapy. Radiat. Oncol..

[5] Budäus L., Bolla M., Bossi A., Cozzarini C., Crook J., Widmark A., Wiegel T. (2012). Functional outcomes and complications following radiation therapy for prostate cancer: a critical analysis of the literature. Eur. Urol..

[6] Montay-Gruel P., Meziani L., Yakkala C., Vozenin M.C. (2018 Apr 25). Expanding the therapeutic index of radiation therapy by normal tissue protection. Br. J. Radiol..

[7] Miyake M., Tanaka N., Hori S., Ohnishi S., Takahashi H., Fujii T., Owari T., Ohnishi K., Iida K., Morizawa Y., Gotoh D., Itami Y., Nakai Y., Inoue T., Anai S., Torimoto K., Aoki K., Fujimoto K. (2019). Dual benefit of supplementary oral 5-aminolevulinic acid to pelvic radiotherapy in a syngenic prostate cancer model. Prostate.

[8] Miyake M., Ishii M., Kawashima K., Kodama T., Sugano K., Fujimoto K., Hirao Y. (2009). siRNA-mediated knockdown of the heme synthesis and degradation pathways: modulation of treatment effect of 5-aminolevulinic acid-based photodynamic therapy in urothelial cancer cell lines. Photochem. Photobiol..

[9] Terada Y., Inoue K., Matsumoto T., Ishihara M., Hamada K., Shimamura Y., Ogata K., Inoue K., Taniguchi Y., Horino T., Karashima T., Tamura K., Fukuhara H., Fujimoto S., Tsuda M., Shuin T. (2013). 5-Aminolevulinic acid protects against cisplatin-induced nephrotoxicity without compromising the anticancer efficiency of cisplatin in rats in vitro and in vivo. PloS One.

[10] Uchida A., Kidokoro K., Sogawa Y., Itano S., Nagasu H., Satoh M., Sasaki T., Kashihara N. (2019). 5-aminolevulinic acid exerts renoprotective effect via Nrf2 activation in murine rhabdomyolysis-induced acute kidney injury. Nephrology.

[11] Takase N., Inden M., Sekine S.I., Ishii Y., Yonemitsu H., Iwashita W., Kurita H., Taketani Y., Hozumi I. (2017). Neuroprotective effect of 5-aminolevulinic acid against low inorganic phosphate in neuroblastoma SH-SY5Y cells. Sci. Rep..

[12] Zhao M., Zhu P., Fujino M., Nishio Y., Chen J., Ito H., Takahashi K., Nakajima M., Tanaka T., Zhao L., Zhuang J., Li X.K. (2016). 5-Aminolevulinic acid with sodium ferrous citrate induces autophagy and protects cardiomyocytes from hypoxia-induced cellular injury through MAPK-Nrf-2-HO-1 signaling cascade. Biochem. Biophys. Res. Commun..

[13] Tanaka N., Asakawa I., Kondo H., Tanaka M., Fujimoto K., Hasegawa M., Konishi N., Hirao Y. (2009). Technical acquisition and dosimetric assessment of iodine-125 permanent brachytherapy in localized prostate cancer: our first series of 100 patients. Int. J. Urol..

[14] Tanaka N., Torimoto K., Asakawa I., Miyake M., Anai S., Hirayama A., Hasegawa M., Konishi N., Fujimoto K. (2014). Use of alpha-1 adrenoceptor antagonists in patients who underwent low-dose-rate brachytherapy for prostate cancer - a randomized controlled trial of silodosin versus naftopidil. Radiat. Oncol..

[15] Abrams P., Chapple C., Khoury S., Roehrborn C., de la Rosette J., International Scientific Committee (2009). Evaluation and treatment of lower urinary tract symptoms in older men. J. Urol..

[16] Roach M., Hanks G., Thames H., Schellhammer P., Shipley W.U., Sokol G.H., Sandler H. (2006). Defining biochemical failure following radiotherapy with or without hormonal therapy in men with clinically localized prostate cancer: recommendations of the RTOG-ASTRO Phoenix Consensus Conference. Int. J. Radiat. Oncol. Biol. Phys..

[17] Tanaka N., Fujimoto K., Hirao Y., Asakawa I., Hasegawa M., Konishi N. (2009). Variations in international prostate symptom scores, uroflowmetric parameters, and prostate volume after (125)I permanent brachytherapy for localized prostate cancer. Urology.

[18] Nakai Y., Tanaka N., Asakawa I., Torimoto K., Miyake M., Anai S., Fujii T., Hasegawa M., Fujimoto K. (2019). Analysis of quality of life after randomized controlled trial of alpha-1 adrenoceptor antagonist alone and in combination with cyclooxygenase-2 inhibitor in patients who underwent low-dose-rate brachytherapy for prostate cancer. J. Contemp. Brachytherapy.

[19] Tanaka N., Asakawa I., Hasegawa M., Fujimoto K. (2015). Urethral toxicity after LDR brachytherapy: experience in Japan. Brachytherapy.

[20] Tanaka N., Torimoto K., Asakawa I., Miyake M., Anai S., Nakai Y Y., Fujii T., Hasegawa M., Fujimoto K. (2018). Comparison of chronological changes in urinary function in patients who underwent low-dose-rate brachytherapy for prostate cancer-A randomized controlled trial of alpha-1 adrenoceptor antagonist alone versus combination with cyclooxygenase-2 inhibitor. Brachytherapy.

[21] Yan M., Xue P., Wang K., Gao G., Zhang W., Sun F. (2017). Does combination therapy with tamsulosin and trospium chloride improve lower urinary tract symptoms after SEEDS brachytherapy for prostate cancer compared with tamsulosin alone?: a prospective, randomized, controlled trial. Strahlenther. Onkol..

[22] Tanaka T., Morimoto K., Nishikawa N., Kuratsukuri K., Ishii K., Yoshimura R., Nakatani T. (2012). Suppressive effects of eviprostat, a phytotherapeutic agent, on lower urinary tract symptoms in prostate cancer patients treated with brachytherapy. Low. Urin. Tract. Symptoms.

[23] Al-Saber F., Aldosari W., Alselaiti M., Khalfan H., Kaladari A., Khan G., Harb G., Rehani R., Kudo S., Koda A., Tanaka T., Nakajima M., Darwish A. (2016). The safety and tolerability of 5-aminolevulinic acid phosphate with sodium ferrous citrate in patients with type 2 diabetes mellitus in Bahrain. J. Diabetes. Res..

[24] Masuki S., Morita A., Kamijo Y., Ikegawa S., Kataoka Y., Ogawa Y., Sumiyoshi E., Takahashi K., Tanaka T., Nakajima M., Nose H. (2016). Impact of 5-aminolevulinic acid with iron supplementation on exercise efficiency and home-based walking training achievement in older women. J. Appl. Physiol..

[25] Atkinson T.M., Ryan S.J., Bennett A.V., Stover A.M., Saracino R.M., Rogak L.J., Jewell S.T., Matsoukas K., Li Y., Basch E. (2016). The association between clinician-based common terminology criteria for adverse events (CTCAE) and patient-reported outcomes (PRO): a systematic review. Support Care Cancer.

[26] Bennett B.K., Park S.B., Lin C.S., Friedlander M.L., Kiernan M.C., Goldstein D. (2012). Impact of oxaliplatin-induced neuropathy: a patient perspective. Support Care Cancer.

